# A Sustainable Adhesive Paradigm: Reversibly Reinforcing, Heat‐Free Bonding with Universal Substrates

**DOI:** 10.1002/advs.202516031

**Published:** 2025-12-08

**Authors:** Shuang Zhang, Xin Jing, Shilong Wu, Fang Liu, Xiaoyuan Li, Yubin Huang

**Affiliations:** ^1^ Faculty of Chemistry Northeast Normal University Changchun 130024 China; ^2^ Changchun Institute of Applied Chemistry Chinese Academy of Science Changchun 130022 China; ^3^ School of Chemistry and Chemical Engineering (Silicon Industry) Inner Mongolia University of Science and Technology Baotou 014010 China

**Keywords:** degradable polymer adhesive, nacre‐inspired structure, repeated and cryogenic adhesive

## Abstract

Non‐biodegradable adhesives accumulate in ecosystems and contribute to contamination. They even contaminate recyclable materials, reducing the efficiency of recycling processes or rendering them impossible. Transitioning to biodegradable or recyclable adhesives is critical to mitigating these environmental impacts and advancing zero‐waste initiatives. A nacre‐inspired hard‐soft multiphase structure is developed to serve as a heat‐free adhesive system, delivering superior adhesion strength across metal, plastic, and glass substrates. Autonomous reconstruction of physically cross‐linked networks and strain‐induced orientation synergistically achieve up to 120 de‐bonding and re‐bonding cycles with progressive adhesion enhancement. Repeatable bonding with reinforcement instead of strength reduction enables error‐tolerant applications. Unique thermal responsiveness that maintains exceptional adhesion performance under low‐temperature conditions, especially in liquid nitrogen environments, is investigated. The dynamic equilibrium of non‐covalent interactions coupled with hydrolytically sensitive ester bonds permits tunable degradation kinetics through compositional modulation. This design paradigm establishes an eco‐friendly alternative to conventional adhesives, particularly suitable for applications demanding recyclability, environmental compatibility, repeated usability, and operation under ultra‐low temperature conditions.

## Graphical abstract

A heat‐free adhesive system, delivering superior adhesion strength across metal, plastic, and glass substrates, is developed. Repeatable bonding with reinforcement instead of strength reduction enables error‐tolerant applications. This design paradigm establishes an eco‐friendly alternative to conventional adhesives, particularly suitable for applications demanding recyclability, environmental compatibility, repeated usability, and operation under ultra‐low temperature conditions.

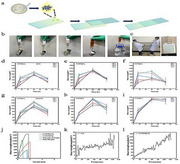

## 1 Introduction

Adhesives are widely used to assemble most consumer goods and industrial products. While modern adhesives provide strong, durable bonds, their ubiquitous application incurs substantial environmental costs, including high energy consumption, limited degradability, and recycling difficulties. Consequently, many bonded products are ultimately discarded, landfilled, or incinerated, exacerbating resource depletion, microplastic pollution, and harmful emissions.^[^ ^1^, ^2^ ^]^ Initial shift from petroleum sourced to environmentally friendly adhesives encountered compromised performance. What's exciting is that current adhesion strengths of these novel adhesives have increased to thousands or even megapascals, exhibiting equivalent or superior adhesive capacity to existing marketed adhesives (such as conventional epoxies).^[^ ^3^, ^4^, ^5^ ^]^ These breakthroughs challenge the traditional assumption that degradability necessitates mechanical compromise, instead proving that high performance and sustainability can coexist. This paradigm shift is driving the development of universally applicable and sustainable adhesives.^[^ ^6^, ^7^ ^]^

Recognized for its exceptional biodegradability and biocompatibility, PLGA‐PEG‐PLGA (polylactic acid‐polyethylene glycol‐polylactic acid) copolymer has been widely utilized in biomedical materials and advanced industrial applications.^[^ ^8^, ^9^, ^10^ ^]^ Notably, it is approved by the FDA as a pharmaceutical excipient and listed in the U.S. Pharmacopoeia.^[^ ^11^ ^]^ Current research predominantly focuses on its use on drug‐loaded nanoparticles or thermosensitive hydrogels, which exhibit sol–gel transitions between room and body temperatures, enabling injectable sustained drug delivery systems.^[^ ^12^, ^13^ ^]^ Intriguingly, we observed that the low‐molecular‐weight PLGA‐PEG‐PLGA copolymer itself displayed inherent viscoelasticity, heat free and inverse thermal transition behavior compared to traditional sol–gel systems. Specifically, the polymer transitioned to a viscous flow state upon heating, whereas cooling drastically reduced mobility and increased viscosity, exhibiting a phenomenon diametrically opposed to that of conventional sol–gel hydrogels. The polyether backbone could offer flexibility, high biocompatibility, and contribute significantly to H‐bonding, particularly as H‐bonding acceptors with other functional groups.^[^ ^14^ ^]^ Although this copolymer adheres well to diverse substrates, its room‐temperature bonding strength and ductility need further improvement to meet application demands. Inspired by the biological example of tough seashell nacre that features a hard‐soft multiphase structure (rigid aragonite hexagonal platelets are interconnected by a soft protein matrix), we developed a dual reinforcement strategy: a chemically crosslinked PLGA‐PEG‐PLGA network serving as the soft matrix, and rigid platelet‐like nanoparticles formed through divalent metal chloride‐DOPA chelation, mimicking nacre's hard phase.^[^ ^15^, ^16^, ^17^, ^18^ ^]^ As a critical component of mussel adhesive proteins, DOPA enhances interfacial adhesion and bulk cohesion through interactions with metal ions and the polymer.^[^ ^19^, ^20^, ^21^, ^22^ ^]^ The developed adhesives demonstrated multifunctional substrate compatibility spanning polymeric, metallic (aluminum alloy), and vitreous surfaces, with the MgCl_2_ incorporated formulation achieving peak shear strength of 2.7 MPa under standard conditions, surpassing conventional Deli universal glue (0.9 MPa), and 502 Glue (1.8 MPa) commercial adhesives by 200% and 50% respectively. In contrast to conventional disposable structural adhesives that rely on oxygen initiated rapid polymerization mechanisms, these newly developed formulations exhibited inherent fault tolerance, exceptional cyclic stability, and reusability through reversible interfacial bonding. Progressive adhesion enhancement was observed, and high lap shear strength after 120 cycles were retained. This behavior outperformed conventional pressure‐sensitive adhesives that typically exhibit rapid strength degradation within a few cycles. Notably, the MgCl_2_ incorporated adhesive system exhibited significantly enhanced viscoelastic properties at −30 °C, with storage modulus (*G*') reaching 1023.3 MPa and complex viscosity (η^*^) attaining 1.02 × 10^9^ Pa·s, representing 10.4 fold and 7.4 fold increases, respectively, compared to pure adhesive. Superior bonding strength was kept even when being immersed into a liquid nitrogen atmosphere. These characteristics make this eco‐friendly adhesive particularly suitable for dynamic medical scenarios requiring periodic replacement (surgical dressings, implantable sensors), next‐generation wearable electronics requiring skin compliance, temporary industrial fixtures in sustainable manufacturing, and specialized bonding operations in polar environments or cryogenic storage systems.

## 2 Results and Discussion

### 2.1 Synthesis and Spectra Characterizations of Adhesives

Since their approval by the US Food and Drug Administration (FDA) for clinical applications, PLGA‐PEG‐PLGA copolymers have been extensively studied owing to their biodegradability. Most current PEG‐PLGA based polymers are commercially available as powdered products and exhibit core–shell structures in aqueous solutions due to their amphiphilic nature. They form thermo‐sensitive hydrogels that remain in a sol state at room temperature but rapidly undergo gelation upon injection into the body.^[^ ^12^, ^23^ ^]^

**Figure 1:**
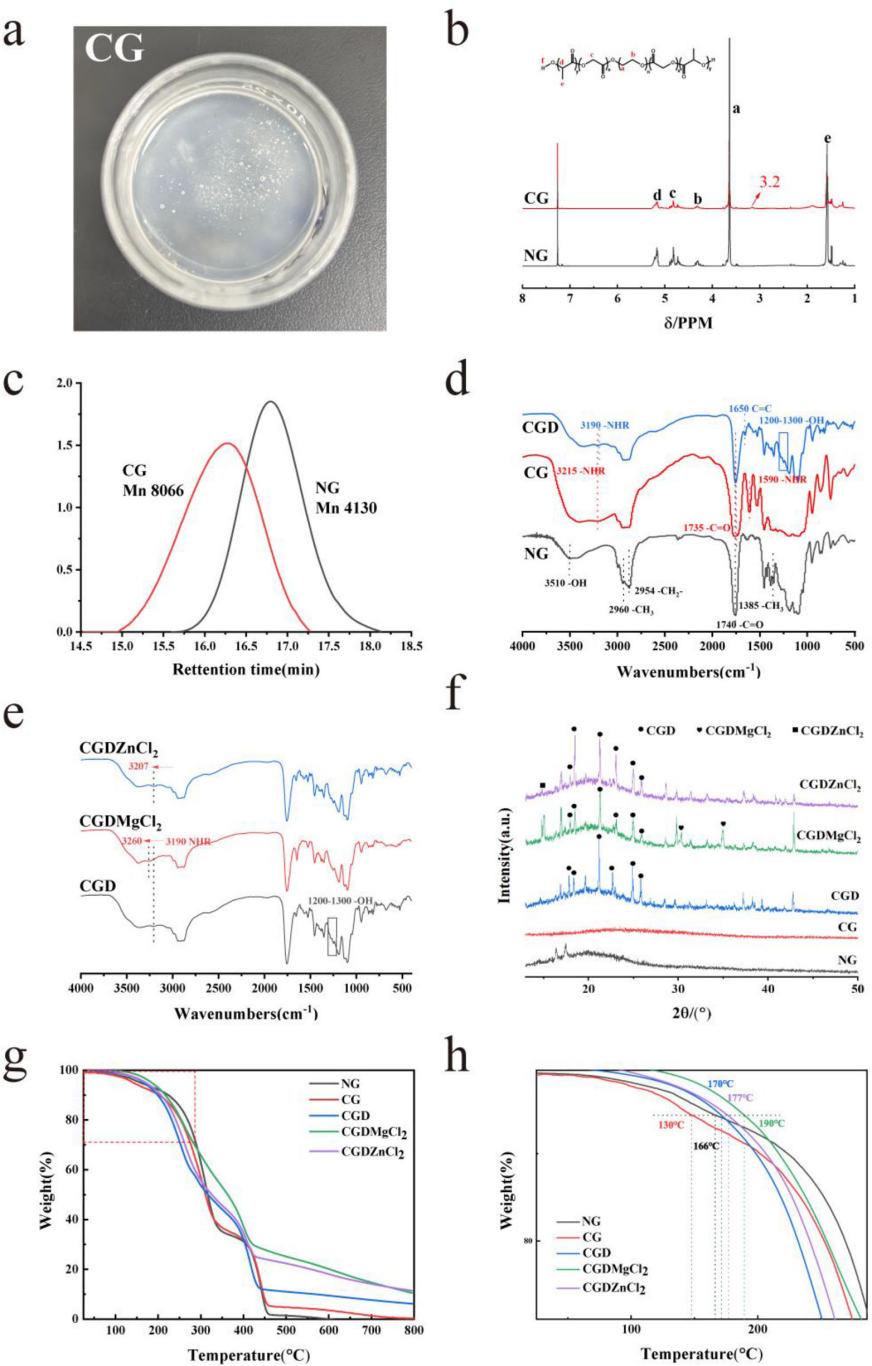
Appearance and characterization of adhesives. a) Appearance of CG. b) ^1^H NMR spectrum of adhesives. c) GPC profiles of NG and CG. d,e) FT‐IR spectra of adhesives. f) XRD patterns of adhesives. g,h) TGA curves of adhesives.

Serendipitously, it was observed that reducing the LA/GA molar ratio to 1.3 and shortening the ring‐opening polymerization reaction time to 6 h yielded a PLGA‐PEG‐PLGA copolymer with significantly lower molecular weight. Following precipitation and vacuum drying, the product itself exhibited a viscous adhesive morphology. To enhance viscosity, HDI served as both a chain extender and primary chemical crosslinker (Figure S1, Supporting Information), achieving 86% reaction yield. The resulting product exhibited an off‐white translucent appearance (**Figure** **1**a). The corresponding ^1^H NMR spectra confirmed the chemical structures and the correspondence to the chemical structures were as follows: *δ* 1.55 as ─CH_3_ of lactic acid, *δ* 3.60 as ─O─CH_2_─ of PEG, *δ* 4.30 as ─CH_2_─ of PEG, *δ* 4.80 as ─CH_2_ of glycolic acid, *δ* 5.05 as ─CH─ of lactic acid and *δ* 3.20 as ─CH_2_─N═C═O of HDI (Figure 1b). The gel permeation chromatography (GPC) results demonstrated that the number‐average molecular weight (M_n_) of the block copolymer PLGA‐PEG‐PLGA (abbreviated as NG) increased significantly from 4130 to 8066 g mol^−1^ after crosslinking with HDI (abbreviated as CG), as illustrated in Figure 1c. Concurrently, the polydispersity index (PDI) exhibited only a slight increment from 1.28 to 1.37, indicating that the crosslinking process had a negligible impact on the molecular weight distribution of the copolymer. This observation suggested that the crosslinking reaction primarily enhanced molecular chain extension without substantially altering the material's homogeneity.

Rigid platelet‐like nanoparticles formed through divalent metal chloride and DOPA chelation were subsequently integrated into the polymer matrix to mimic nacre's hard phase. For comparative analysis, NG, CG, and CGD (CG blended with L‐dopa) were systematically fabricated as control samples. A comprehensive suite of analytical techniques was employed to elucidate the chemical and physical bonding mechanisms within this composite material.

The FT‐IR spectral analysis of NG, CG, CGD, and CGDXCl_2_ samples (Figure 1d,e) revealed critical structural information: a distinct absorption band at 1740 cm^−^^1^ confirmed C═O stretching vibrations from ester groups formed during ring‐opening polymerization. Characteristic PLA vibrations appeared at 1385 and 2960 cm^−^^1^, while PGA exhibited its signature stretching vibration at 2954 cm^−^^1^. Wide adsorption band at 3215 cm^−1^ and sharp peak at 1590 cm^−1^ in CG could be attributed to the stretching vibrations of secondary amine. Diagnostic absorption at 1650 cm^−^^1^ that corresponded to the stretching vibration of C═C in aromatic ring verified successful L‐dopa integration into the polymer matrix. Intensity of the sharp peak at 1590 cm^−1^ decreased a lot in comparison with CG, and another shift from 3215 to 3190 cm^−1^ confirmed the hydrogen bonding formation between L‐dopa and polymer. Significant hydroxyl stretching shifts were observed with the incorporation of divalent metal chlorides (from 3190 cm^−1^ for CGD to 3260 cm^−1^ for CGDMgCl_2_ and 3205 cm^−1^ for CGDZnCl_2_). These hypsochromic shifts, proportional to metal ion electronegativity, demonstrated metal‐dopa coordination complex formation through electron density withdrawal from phenolic ─OH groups.^[^ ^24^ ^]^ Similar coordination behavior was observed across all the other divalent metal chloride‐modified adhesives (Figure S2, Supporting Information).

X‐ray diffraction (XRD) study was conducted to trace the composition process (Figure 1f). Elimination of native diffraction peaks at 16.41° and 17.51° (2θ) and disappearance of the broad semicrystalline peak of NG indicated HDI crosslinking induced crystallinity suppression or disruption of partial ordered packing. With the addition of L‐dopa, diagnostic diffraction peaks at 17.95°, 18.40°, 21.25°, 22.75°, 24.95°, and 25.90° were detected.^[^ ^25^ ^]^ Composite adhesives exhibited distinct inorganic phase signatures. Distinctive peaks at 2θ 30.28° and 34.93° were ascribed to MgCl_2_. Typical peaks ascribed to copolymer, L‐dopa, and MgCl_2_ were all found with CGDMgCl_2_. The characteristic peak corresponding to ZnCl_2_ at 16.26° also confirmed the formation of the composite CGDZnCl_2_ adhesive. Other divalent metal chlorides were clearly ascribed, respectively, as shown in Figure S3 (Supporting Information), and the peaks were in good consistency with the XRD standard card. Notably, re‐emergence of PLGA‐PEG‐PLGA matrix signature and recovery of semi‐crystallinity revealed that incorporation of L‐dopa and divalent metal chlorides facilitated significant structural reorganization through chelation‐induced chain alignment and establishment of h hydrogen bonding networks.

The thermal stability profiles of all adhesive materials were evaluated using thermogravimetric analysis (TGA) under a nitrogen atmosphere. As shown in Figure 1g,h, the initial weight loss observed across all adhesive samples (2–5% mass decrease between 50 and 150 °C) corresponded to the evaporation of absorbed moisture and residual water molecules from environmental exposure, a characteristic phenomenon commonly associated with hygroscopic polymeric materials. TGA revealed distinct thermal decomposition characteristics among the adhesive samples, with 5% weight loss (T5%) occurring at 130 °C for CG, 170 °C for CGD, 190 °C for CGDMgCl_2_, and 177 °C for CGDZnCl_2_. Synergistic effects of L‐dopa and metal chloride additives demonstrated a stepwise enhancement in thermal stability, suggesting the cooperative contribution of hydrogen‐bonding networks and metal‐chelation interactions. Particularly in the case of CGDMgCl_2_, which exhibited superior thermal resistance with the highest decomposition temperature, achieving significant improvements compared to CG and CGD, respectively. This phenomenon correlated with its significantly reduced maximum decomposition rate as evidenced by derivative thermogravimetric (DTG) analysis (Figure S4a, Supporting Information), along with the highest char residue retention (32.1% at 800 °C), confirming its optimal thermal stability among the tested formulations. The complete thermal decomposition profiles of other modified adhesive formulations are presented in Figure S4b,c (Supporting Information).

**Figure 2:**
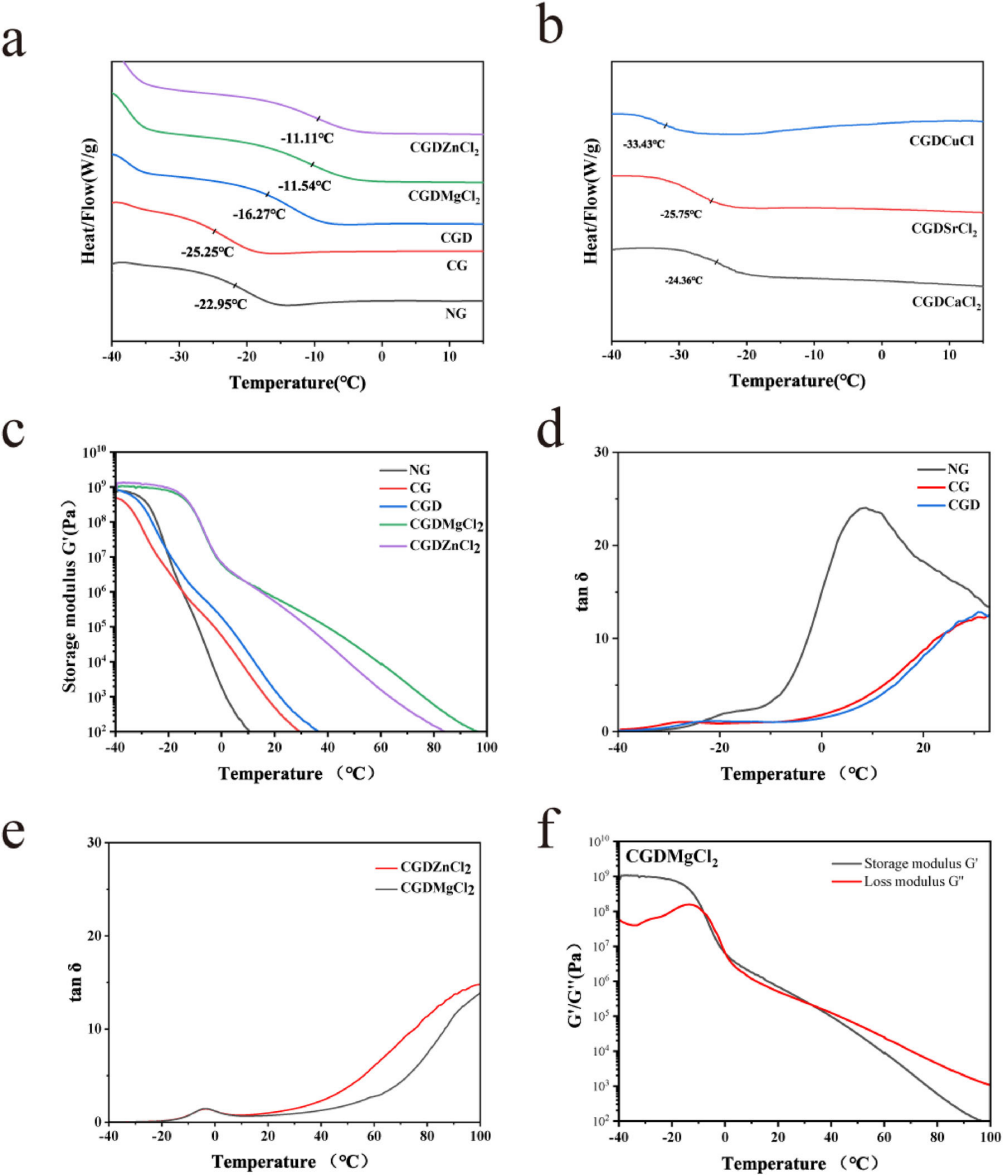
Rheological curves. a,b) DSC curves of adhesives. c) Storage modulus of the adhesives as a function of temperature. d,e) Temperature‐dependent tan δ curves of the adhesive formulations. f) Storage modulus and loss modulus of CGDMgCl_2_ across varying temperatures.

Differential Scanning Calorimetry (DSC) analysis revealed significant evolution in the viscoelastic properties of adhesive formulations (**Figure** **2**a,b). The base NG adhesive exhibited a glass transition temperature (*T*g) of −22.95 °C, which decreased to −25.25 °C after HDI crosslinking (CG), suggesting a potential increase in free volume within the polymer network. Remarkably, L‐dopa incorporation (CGD) induced a dramatic *T*g elevation to −16.27 °C. This enhancement was attributed to dual mechanisms: 1) aromatic moieties enhancing the rigidity of the cross‐linked network through restricted chain mobility; 2) catechol‐mediated secondary crosslinking via hydrogen bonding interactions, as supported by FT‐IR spectroscopic analysis.^[^ ^26^ ^]^ Another key factor of metal chelation originated from divalent metal chlorides working together with L‐dopa to increase the *T*g of CGDMgCl_2_ to −11.54 °C and CGDZnCl_2_ to −11.11 °C. The glass transition temperatures of the adhesives were all below 0 °C, indicating that their molecular chains and chain segments could move freely (Figure S4d, Supporting Information). This resulted in a viscous flow state under ambient conditions, which endowed the materials with intrinsic repair capability without requiring solvent activation.

Rheological characterization was conducted to evaluate the viscoelastic response of all formulations under forced sinusoidal deformation. Key parameters, including storage modulus *G*' (elastic energy storage capacity), loss modulus *G*'' (viscous energy dissipation via flow and intermolecular friction), loss factor tan δ (*G*''/*G*''), and complex viscosity, were monitored during broad‐range temperature sweeps. At sub‐*T*g temperatures, all systems behaved as elastomeric binders, transitioning from glassy to rubbery viscous flow states upon heating (Figure 2c). The observed transition temperatures (−17.95 °C for NG, −27.36 °C for CG, −23.25 °C for CGD, −2.87 °C for CGDMgCl_2_, and −3.20 °C for CGDZnCl_2_) showed strong correlation with DSC data, manifesting as primary tan *δ* peaks corresponding to molecular rearrangement events (Figure 2d,e). Notably, despite HDI crosslinking (CG) increasing network density, its linear chain segments created additional free volume at low temperatures, thereby enhancing polymer chain mobility.^[^ ^27^ ^]^ This molecular plasticization effect resulted in a transition temperature depression relative to NG from −17.95 to −27.36 °C. Partial recovery of transition temperature to −23.25 °C in CGD stemmed from L‐dopa's hydrogen bonding with the polymer matrix. Remarkably, metal chloride incorporation (CGDMgCl_2_: −2.87 °C; CGDZnCl_2_: −3.20 °C) induced obvious transition temperature elevation, directly evidencing metal‐catechol coordination's restrictive effects on chain mobility (Figure 2e).

The second peaks in the tan *δ* curves of these adhesives exhibited intensity trends similar to the first peak and were attributed to mobility‐restricted regions primarily associated with crosslinked domains.^[^ ^27^ ^]^ Notably, NG displayed the highest peak intensity and the lowest corresponding temperature, suggesting enhanced molecular mobility and reduced cohesive energy density compared to modified formulations. Upon incorporating L‐dopa and XCl_2_, a significant increase in crosslinking density υ_e_ at room temperature (calculated from plateau *G*' values, as shown in Table S2, Supporting Information) was observed, accompanied by a notable temperature shift of the secondary tan *δ* peaks to higher values.

The temperature‐dependent viscoelastic behavior was evidenced by the progressive decrease in both *G*' and G'' with increasing temperature, indicative of thermally reversible adhesive properties (Figure 2f; Figure S5, Supporting Information). This thermally sensitive behavior and changing trend mirror those of the supramolecular polymeric adhesive prepared via water‐mediated hydrogen bonding and the inorganic subnanometer nanowire (SNW) adhesive, both achieving high strength and great reversibility through multiple physical and weak interactions.^[^ ^6^, ^28^ ^]^ The low‐temperature‐triggered strengthening behavior positioned these materials as promising candidates for ultra‐low temperature applications, particularly in Arctic/Antarctic environments or space exploration scenarios where conventional hot‐melt adhesives often fail to maintain stable bonding.^[^ ^29^ ^]^ Remarkably, XCl_2_‐modified adhesives demonstrated superior mechanical reinforcement across the entire temperature spectrum investigated. For CG and CGD samples, no rubbery plateau was observed during the temperature sweep, indicating the absence of physical crosslinking in the system. However, after the addition of XCl_2_, the modified adhesives exhibited a rubbery plateau in the rheological temperature sweep, suggesting the formation of physical crosslinking in the system. The similar plateau modules indicated that the association density within the system was comparable. Notably, the MgCl_2_ modified adhesives showed a broader plateau and a higher solid‐liquid transition temperature compared to the ZnCl_2_ modified adhesives. This is attributed to the cation radius (rZn > rMg), which leads to stronger physical association activation energy in the MgCl_2_‐modified adhesives. On the other hand, this inverse correlation also suggested that adhesive strength deteriorated at elevated temperatures, enabling facile thermal de‐bonding through mild heating. These thermally responsive “smart” characteristics position these adhesives as sustainable switchable bonding solutions, particularly promising for: programmable robotics requiring localized motion control; circular manufacturing processes with reversible assembly/disassembly; eco‐conscious material systems demanding reuse and recycling capabilities. This work successfully reconciled the conventionally conflicting requirements of high‐strength bonding and non‐destructive de‐bonding through precise thermal modulation.^[^ ^30^, ^31^ ^]^ The seemingly paradoxical properties of high adhering strength and reuse could well be combined.^[^ ^32^ ^]^

### 2.2 Surface Hydrophilicity and Morphology

**Figure 3:**
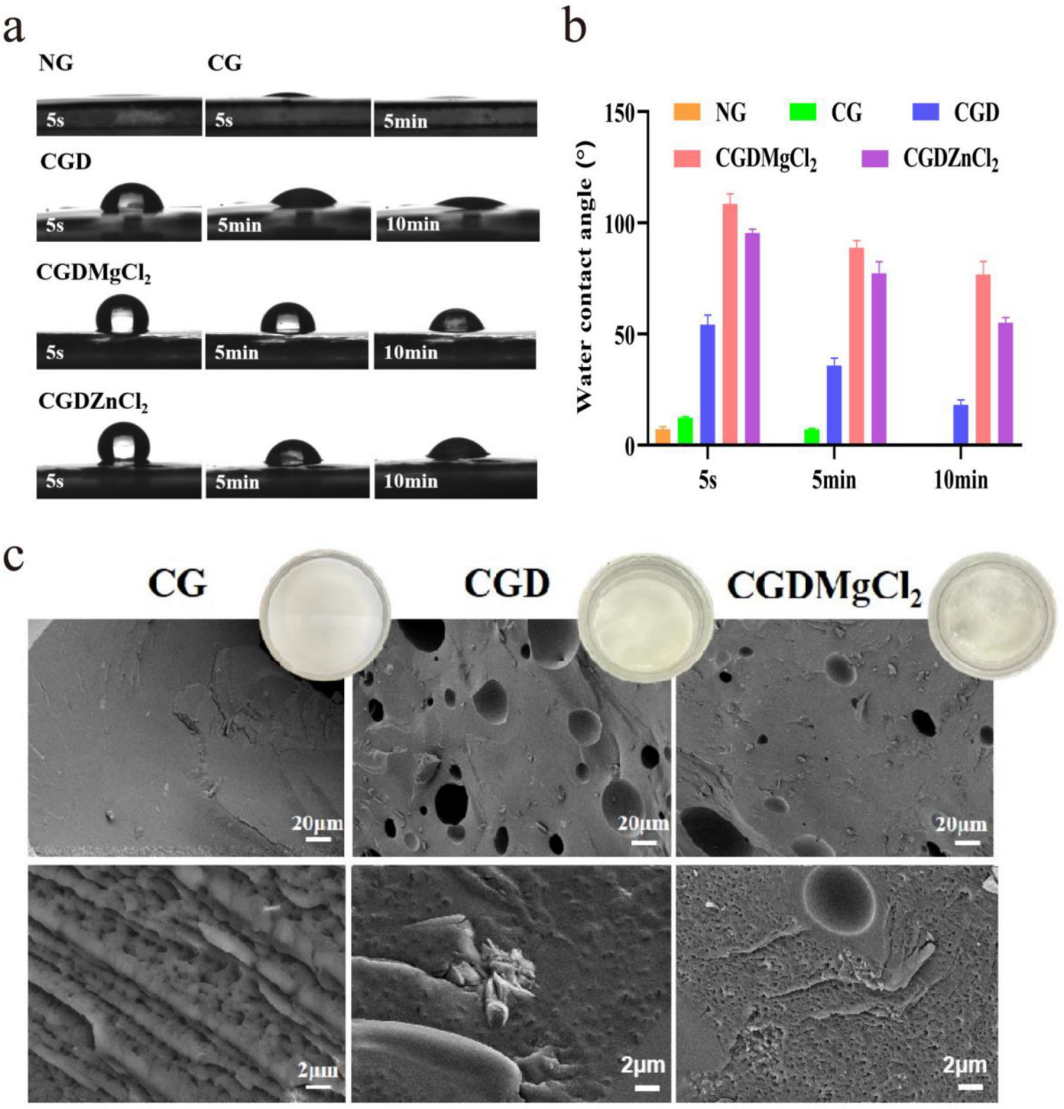
Hydrophilicity and microstructure. a,b) Water contact angle measurement. c) The microstructure of the CG, CGD, and CGDMgCl_2_ adhesives.

Contact angle measurements quantitatively validated the modification effects. NG achieved complete adsorption of the water droplet within 40 s, whereas CG required a longer duration. Adsorption kinetics were further suppressed in CGD and CGDXCl_2_, likely due to their crosslinked networks and restricted molecular mobility (**Figure** **3**a,b; Figure S6, Supporting Information). This prolonged reduction in contact angle correlated directly with bulk adhesive performance metrics. Collectively, these findings confirm the establishment of strong chemophysical interactions at the molecular level, consistent with the proposed crosslinking mechanism in Figure S1 (Supporting Information).^[^ ^33^, ^34^ ^]^

The microstructure‐property relationship of adhesives was investigated using Cryo‐SEM (Figure 3c). Macroscopically, CG exhibited homogeneous, defect‐free surface morphology. High‐resolution imaging revealed an interconnected crosslinked network featuring periodic ridge structures and uniformly distributed micropores, attributed to the primary crosslinking induced by HDI. The incorporation of L‐dopa induced significant morphological alterations, such as macrospore formation with uniform L‐dopa encapsulation. Hierarchical porosity is supposed to evolve through three mechanisms: 1) oxidative conversion of phenolic hydroxyl groups into quinone or semi‐quinone, which continuously produces hydrogen peroxide and subsequently decomposes to generate oxygen and water;^[^ ^35^ ^]^ 2) enhanced hydrogen‐bonding networks between L‐dopa's amino/quinone functionalities and the CG matrix, facilitating water molecule aggregation; 3) freeze‐drying‐induced water sublimation during sample processing. Notably, the addition of MgCl_2_ preserved the matrix architecture while introducing nanoscale clusters and acted as a platelet‐like reinforcing hard phase. These nanoclusters roughened final interfaces and dissipated energy and functioned as oxygen reservoirs by trapping and gradually releasing atmospheric oxygen into the adhesive bulk, thereby accelerating the phenol‐quinone oxidative reactions and phenol‐amine crosslinking.^[^ ^3^, ^36^, ^37^, ^38^ ^]^ Consequently, additional micropores formed internally, and these architectural features may collectively serve as effective energy dissipation centers that mitigated crack propagation through strain redistribution.^[^ ^39^ ^]^

### 2.3 Adhesion Performance

**Figure 4:**
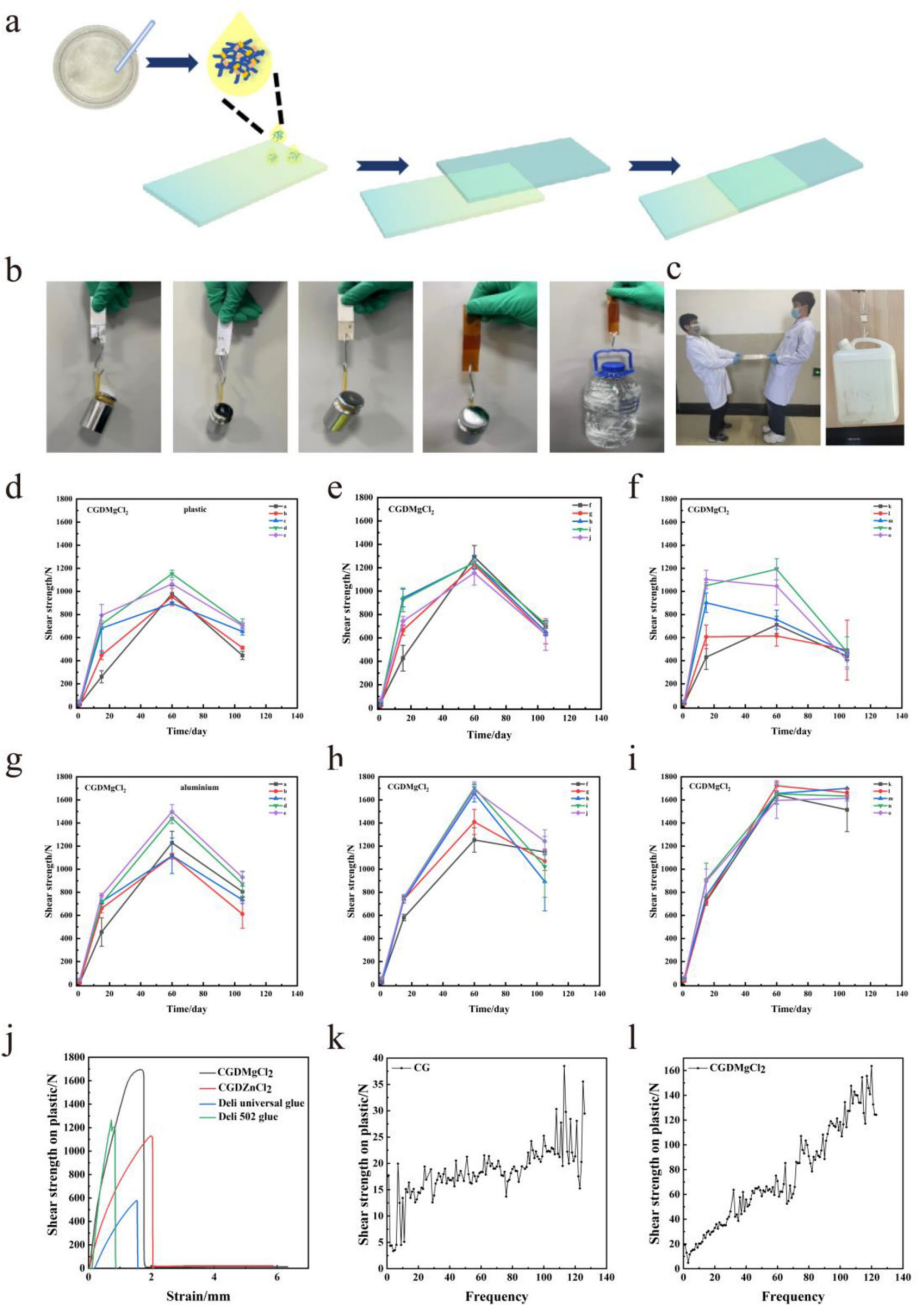
a) Preparation of lap‐shear adhesive samples. b) Photographs of adhesive bonding of various materials (aluminum, cloth, paper, plastic film (PI)). c) Two adult males did not pull apart the CGDMgCl_2_ bonded (25 cm × 25 cm) plastic sheet, and a 2.5 cm^2^ bonded plastic patch can support a 10 kg bucket of water. d–f) Shear strength of the a‐o component of CGDMgCl_2_ on plastic substrates (from Figure 5d–f, the amount of L‐dopa varied from 1 to 2 g and 4 g per 1 g of CG). g–i) Shear strength of the a‐o component of CGDMgCl_2_ on aluminum substrates (from Figure 5g–i, the amount of L‐dopa varied from 1 to 2 g and 4 g per 1 g of CG). j) Comparison of shear strength among CGDMgCl_2_, CGDZnCl_2_, Deli universal glue, and Deli 502 glue on aluminum substrates. k) The evolution of shear strength with increasing bonding cycles (up to 120 times) of CG on the plastic substrate. l) The evolution of shear strength with increasing repeated bonding cycles of CGDMgCl_2_ on plastic substrates.

To evaluate practical applications, the composite adhesive was used to bond various substrates, including aluminum, cloth, hard cardboard, and polyimide (PI) film (**Figure** **4**a,b). Despite a small overlap and adhesion area of only 1 cm^2^, the bonded materials all withstood a 500 g load. In a more demanding test, the adhesive was used to bond PI films, which were then able to lift a 3.5 L bucket of water (≈3.5 kg). Furthermore, in a large‐scale adhesion test with an overlap area of 25 cm × 25 cm, two adult males were unable to pull apart the plastic sheets bonded with the CGDMgCl_2_ adhesive, and a 6.25 cm^2^ bonded plastic patch can support a 10 kg bucket of water (Figure 4c; Movie S1, Supporting Information). These results highlight the exceptional bonding strength and versatility of the composite adhesive for diverse applications.

Lap‐shear tests were conducted to quantify the adhesive strength of the composite materials, with tensile testing providing reproducible data across large sample volumes. From Figure 4d–f, it could be found that for plastic substrates adhered with CGDMgCl_2_, the maximum shear strength increased with L‐dopa content, ranging from 896–1160 N (low L‐dopa, groups a–e) to 1150–1300 N (medium L‐dopa, groups f–j). However, excessive L‐dopa (groups k–o) reduced strength to 610–1190 N, indicating that a medium amount of L‐dopa was sufficient for the elevation of shear strength; While for aluminium substrates, higher L‐dopa and MgCl_2_ contents significantly improved performance (Figure 4g–i). At the 60‐day time point, groups i, j, and l (high L‐dopa/MgCl_2_) achieved the highest shear strengths of 1698–1722 N. Adhesives containing ZnCl_2_ exhibited similar trends: elevated L‐dopa and ZnCl_2_ concentrations typically enhanced shear strength on both substrates. However, group e on aluminum displayed an anomalous increase, deviating from the general pattern (Figure S7a–f, Supporting Information).

Based on the previous findings that medium to high amounts of L‐dopa generally correlate with higher shear strength, additional tests (groups h–m) were conducted on CGDCaCl_2_, CGDSrCl_2_, and CGDCuCl_2_ formulations (Figure S7g–l, Supporting Information). CGDCaCl_2_ exhibited shear strength comparable to CGDZnCl_2_. Notably, its shear strength showed minimal decline at the 105‐day time point and even increased slightly in some cases, indicating excellent long‐term stability. Other formulations also significantly improved adhesion compared to NG, CG, and CGD. However, their strengthening potentials were inferior to MgCl_2_, ZnCl_2_, and CaCl_2_, suggesting limited efficacy in enhancing shear strength.

Shear strength comparisons of CGDMgCl_2_, CGDZnCl_2_, Deli universal glue, and 502 glue on aluminum, glass, and plastic substrates are shown in Figure 4j and Figure S8 (Supporting Information). CGDMgCl_2_ demonstrated superior shear strength compared to CGDZnCl_2_, commercial Deli universal glue, and 502 glue. The commercial adhesives exhibited inherent brittleness, increasing the abrupt failure risk. In contrast, CGDMgCl_2_ and CGDZnCl_2_ dissipated mechanical stresses while simultaneously enhancing strength and ductility, resulting in significant performance improvements.

In contrast to conventional non‐reusable adhesives, our formulations exhibited progressively enhanced shear strength through 120 de‐bonding and re‐bonding cycles (Figure 4k,l; Figure S9, Supporting Information). Especially, the adhesive strength of CGDMgCl_2_ increased up to 8 times of its initial value. This exceptional performance arises from reversible hydrogen bonding and metal‐chelation, enabling repeatable bonding and mechanical enhancement through sacrificial bonds.^[^ ^40^, ^41^, ^42^, ^43^, ^44^ ^]^

Furthermore, performance validation of CGDMgCl_2_ in liquid nitrogen environments confirmed exceptional bonding durability, with adhered glass slides sustaining 500 g loads throughout complete nitrogen immersion cycles (Figure S10 and Movie S2, Supporting Information). The unique structures and noncovalent interactions, which could enhance adhesion and cohesion, collectively overcame key limitations of conventional heat‐melting adhesives, including volumetric shrinkage during freezing, brittle fracture behavior, and impaired stress transfer at polymer‐substrate interfaces.^[^ ^29^, ^45^ ^]^ These features enable flexible, anti‐freezing properties while maintaining strong adhesion at cryogenic temperatures.

### 2.4 Molecular Dynamics Simulations

**Figure 5:**
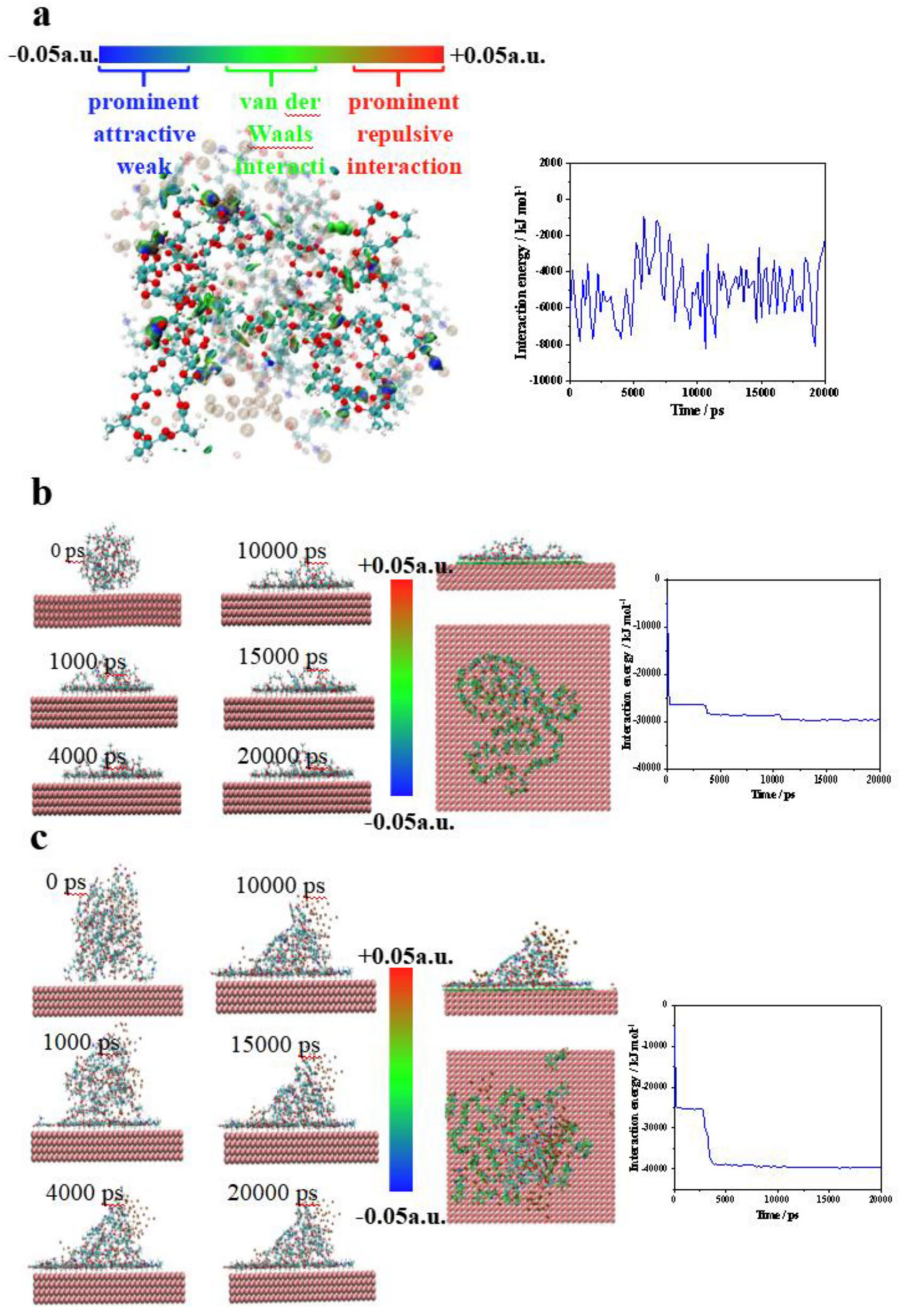
a) IGM analysis of cohesion energy in CGDMgCl_2_ composite adhesive. b) MD and IGM modulation of CGD adherence on aluminium sheet. c) MD and IGM modulation of CGDMgCl_2_ adherence on aluminium sheet.

Strong adhesion relies on two critical factors: cohesion (intra‐adhesive interactions) and surface adhesion (interactions at adhesive‐substrate interfaces). This study employed computational methods to elucidate the molecular mechanisms underlying the performance of the CGDMgCl_2_ adhesive system.^[^ ^46^ ^]^ Cohesion Analysis via IGM analysis was first conducted to determine the aggregation dynamics of CGDMgCl_2_ adhesive. The molecular model system included PLGA‐PEG‐PLGA and HDI as one integrity, L‐dopa and MgCl_2_ as another part. Under simulated conditions, these components aggregated quickly, driven by van der Waals forces (visualized as green regions in IGM) and hydrogen bonds (blue regions) (**Figure** **5**a). The cohesive interaction energy stabilized at −3896.986 kJ mol^−1^, indicating robust and stable intermolecular interactions within the adhesive matrix.

Molecular dynamics (MD) simulations were performed to evaluate the interfacial adhesion of CG and CGDMgCl_2_ on aluminum (Al). A reasonable interaction configuration with minimal energy and a stable state was optimized. After 20 ns of simulation, both CG and CGDMgCl_2_ clusters spread uniformly across the Al surface, demonstrating efficient adsorption. Large amounts of Van De Waals force (green) were observed, and CGDMgCl_2_ exhibited a larger contact area than CG (confirmed by IGM front/vertical views), attributed to enhanced interfacial interactions (Figure 5b,c).

During the initial stage (0–5 ns), weak interaction energies were observed due to the limited contact area caused by the aggregated cluster morphology. Subsequently, in the equilibrium stage (5–20 ns), the clusters dispersed rapidly, maximizing substrate contact. The final interaction energies reached −29690.62 kcal mol^−1^ for CG and −39581.04 kcal mol^−1^ for CGDMgCl_2_. The 33% higher interaction energy of CGDMgCl_2_ compared to CG highlighted the critical role of MgCl_2_ in strengthening adhesion.

### 2.5 Fracture Mechanism of Composite Adhesives

**Figure 6:**
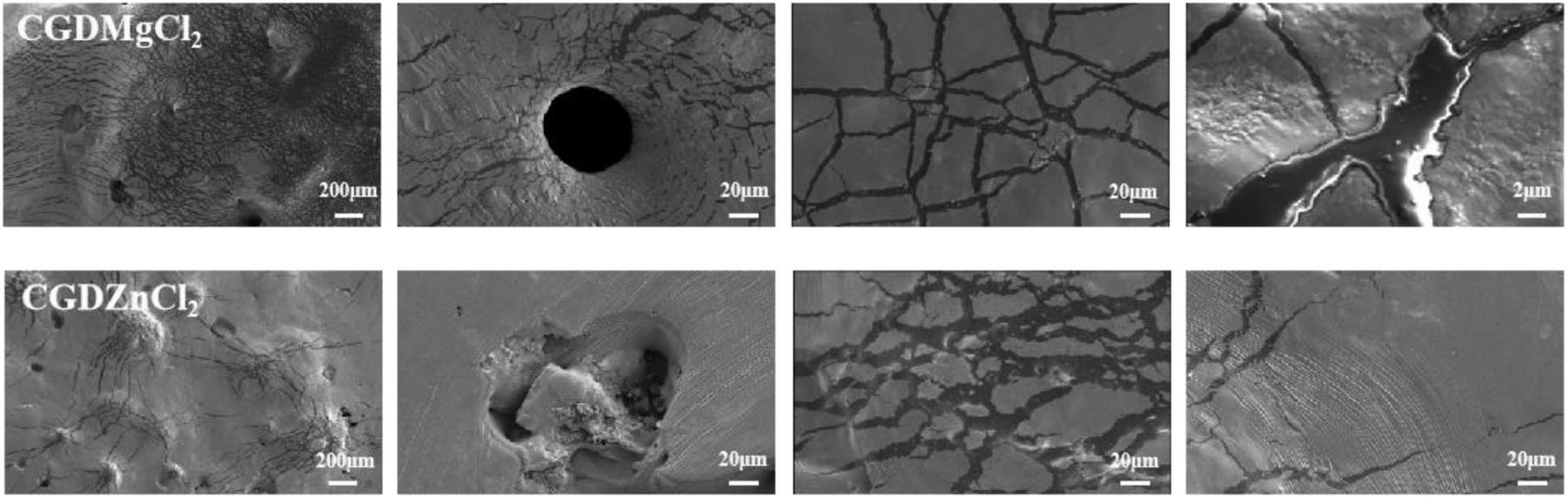
SEM micrographs of the tensile fracture surfaces for CGDMgCl_2_ and CGDZnCl_2_.

The fracture patterns of MgCl_2_ and ZnCl_2_ based adhesives were systematically analyzed using SEM to elucidate their energy dissipation mechanisms and reinforcement strategies (**Figure** **6**). Complex fracture behaviors were observed, including crack deflection and branching, interfacial delamination, cavitation nucleation, and plastic flow. The processes of crack deflection and branching significantly slowed down crack propagation, alleviated and redistributed localized high stresses, and thereby effectively stabilized crack growth.^[^ ^47^ ^]^ Interfacial delamination was primarily attributed to the presence of strong intermolecular sacrificial bonds and interfacial interactions. Notably, MgCl_2_ modified adhesives exhibited a hierarchical fracture analogous to biological seashell nacre in which aragonite platelets serve as “bricks” and are held together by protein “mortar”.^[^ ^48^ ^]^ Rupture of noncovalent bonds distributed stresses, while nanoparticle clusters extracted from the matrix during fracture enabled substantial energy dissipation. This endowed the material with exceptional strength and toughness, preventing complete system failure while enhancing mechanical robustness.^[^ ^49^ ^]^ The biomimetic energy dissipation was further amplified through cavitation nucleation at pore interfaces and plastic deformation of polymer ligaments, synergistically improving fracture toughness via multiscale dissipation mechanisms. Moreover, the development of a fibrillar pattern or striated surface texture is a recognized signature of strain induced alignment in polymer networks. This macroscopic anisotropy may result from the alignment of polymer chains and the reorganization of dynamic bonds along the tensile axis. The correlation between such surface morphology and molecular‐scale orientation has been well established in polymer deformation studies, particularly under significant extensional stress.^[^ ^50^ ^]^ It can be inferred that this progressive alignment during repeated shear tensile cycles facilitated a more efficient distribution of mechanical stress throughout the material. Consequently, greater shear stress was required to cause failure in subsequent cycles. This reinforcement process was consistent with the understanding that strain induced orientation and crystallization were primary enhancing mechanisms in semi‐crystalline polymers.^[^ ^51^, ^52^ ^]^ Collectively, these microstructural features, which were integrated with the dissipative network, delayed catastrophic failure and enabled concurrent energy dissipation and mechanical robustness enhancement in polymer nanocomposite adhesives.

### 2.6 Sealant Application

Adhesive performance in high‐moisture environments is critical for practical applications. Commercial universal adhesives often fail under such conditions, with bonded substrates rapidly fall apart after brief water submersion. In contrast, the developed adhesives exhibited superior performance. They instantaneously adhered to leaking points, sealed them, and prevented water leakage (Figure S11a,b, Supporting Information). Critically, this robust sealing integrity persisted under varying temperatures and pressures (Figure S11c,d, Supporting Information).

### 2.7 Controllable Degradation and Recycle

Controlled or on‐demand degradation is a critical feature for environmentally friendly adhesives. The degradation process was monitored by quantifying the weight loss of adhesives soaked in a PBS buffer solution. Results showed that NG and CG degraded quickly after a few days of immersion (Figure S12a, Supporting Information). This degradation mechanism aroused from the hydrolysis of ester bonds within the polymer backbone, leading to chain scission, gradual reduction in molecular weight, and eventual decomposition into small molecules such as lactic acid (LA), glycolic acid (GA), and ethylene glycol (EG), as confirmed by mass spectrometry (Figure S13, Supporting Information). It was worth noting that CGD exhibited significantly slower degradation, reaching only 43% weight loss by day 311 (Figure S12b, Supporting Information), far below the rates of NG and CG. This enhanced stability in humid environments was attributed to stronger hydrogen bonding and increased crosslinking density facilitated by L‐dopa modifications (Table S2, Supporting Information). Previous studies highlighted that adjusting the ratio of physical crosslinks via cytosine‐mediated hydrogen bonding and end‐group substitutions enabled precise control over stress dissipation, relaxation dynamics, and degradation behavior without altering the polymer backbone.^[^ ^43^ ^]^ Degradation rates also varied across MgCl_2_ containing formulations. For example: CGDMgCl_2_‐h achieved 80% degradation after 234 days, while CGDMgCl_2_‐k degraded nearly completely within 15 days. PH measurements revealed that the higher MgCl_2_ content in the k‐component elevated alkalinity, accelerating ester bond hydrolysis (Figure S12c,d, Supporting Information), as ester bonds are inherently labile in alkaline conditions. These tunable degradation profiles allow customization for diverse applications by adjusting component ratios and corresponding pH values. Furthermore, the adhesives can be deactivated and completely removed via immersion in alcohol solutions without any residue or damage on the substrates, enabling recycling and supporting sustainable use (Figure S12e, Supporting Information).

### 2.8 Biocompatibility of Adhesives

Biocompatibility was evaluated to ensure the safety of the adhesives.^[^ ^53^ ^]^ As shown in Figure S10a–d (Supporting Information), L929 fibroblast viability remained above 80% when exposed to NG, CG, CGD, and CGDMgCl_2_ at concentrations no more than 10 mg mL^−1^ for 24–72 h, confirming their excellent biocompatibility. CGDZnCl_2_ exhibited concentration‐dependent cytotoxicity above 10 mg mL^−1^ within 24 h (Figure S14a–c, Supporting Information), attributable to the inherent ion toxicity of Zn^2^⁺ species.^[^ ^50^ ^]^ Live/dead cell staining images after 24 h’ co‐culture revealed preserved cellular morphology and spreading behavior with negligible dead cells in NG, CG, CGD, and CGDMgCl_2_ groups (Figure S14d, Supporting Information). Morphological abnormalities and cell shrinkages were present in CGDZnCl_2_ groups at high concentration, correlating with MTT assay results. These findings confirmed the exceptional cytocompatibility of NG, CG, CGD, and CGDMgCl_2_ adhesives while highlighting the need for Zn^2^⁺ concentration optimization in biomedical applications. The robust cell compatibility would support their potential for medical applications.

## 3 Conclusion

Environment friendly adhesives of high strength have demonstrated significant potential for sustainable and degradable applications. Our strategy enabled the development of biocompatible, biodegradable adhesives with high performance and tunable degradation rates, leveraging a PLA‐PEG‐PLA (polylactic acid‐polyethylene glycol‐polylactic acid) copolymer backbone. Dynamic reversible hydrogen bonding and metal‐chelation interactions facilitated repeatable adhesion and recyclability, minimizing material waste. Furthermore, these adhesives exhibited versatility in bonding diverse substrates (e.g., plastics, metals, biological tissues) and functioned effectively at extremely low temperatures (even liquid nitrogen atmosphere). Good biocompatibility has established them as candidates for biomedical applications, notably wound closure and tissue engineering. The low‐cost polymer synthesis and straightforward mixing process suggest feasibility for large‐scale industrial production, accelerating the transition toward eco‐friendly adhesive solutions.

## Conflict of Interest

The authors declare no conflict of interest.

## Supporting information



Supporting Information

Supplemental Movie 1

Supplemental Movie 2

## Data Availability

The data that support the findings of this study are available from the corresponding author upon reasonable request.
