# Supplementary material for: A Sustainable Adhesive Paradigm: Reversibly Reinforcing, Heat‐Free Bonding with Universal Substrates
Source: Adv Sci (Weinh). 2025 Dec 8;13(9):e16031. doi: 10.1002/advs.202516031 (PMC12903972; doi:10.1002/advs.202516031)
Supplement: Supplementary file 1 — Supporting Information [file ADVS-13-e16031-s002.doc]

Supporting Information for

**A Sustainable Adhesive Paradigm: Heat-Free Self-Adhesives with Cryogenic-Enhanced, Self-Reinforcing and Universal Bonding**

Shuang Zhang^†^, Xin Jing^†^, Shilong Wu^‡^, Fang Liu^§^, Xiaoyuan Li^*,†^, Yubin Huang^*,†^.

^†^Faculty of Chemistry, Northeast Normal University, China.

^‡^Changchun Institute of Applied Chemistry, Chinese Academy of Science, China.

^§^School of Chemistry and Chemical Engineering (Silicon Industry), Inner Mongolia University of Science and Technology, China.

Corresponding author email: [lixy849@nenu.edu.cn](mailto:lixy849@nenu.edu.cn), [huangyb350@nenu.edu.cn](mailto:huangyb350@nenu.edu.cn).

Table of Contents

[Materials and Methods 3](#__RefHeading___Toc204160188)

[Materials and Instrumentation 3](#__RefHeading___Toc204160189)

[Methods 3](#__RefHeading___Toc204160190)

[Preparation of Adhesives 3](#__RefHeading___Toc204160191)

[Characterization of Adhesives 4](#__RefHeading___Toc204160192)

[Molecular Dynamics Simulations 5](#__RefHeading___Toc204160193)

[Sealant Test 6](#__RefHeading___Toc204160194)

[In Vitro Degradation 6](#__RefHeading___Toc204160195)

[Biocompatibility of Adhesives 7](#__RefHeading___Toc204160196)

[Statistical Analysis 7](#__RefHeading___Toc204160197)

[References 7](#__RefHeading___Toc204160198)

[Supplementary Figures and Tables 8](#__RefHeading___Toc204160199)

[Movie Descriptions 17](#__RefHeading___Toc204160200)

# Materials and Methods

## Materials and Instrumentation

Poly (ethylene glycol) (PEG Mn = 1500 g/mol), glycolide acetate (GA), d,l-lactide acetate (LA), hexamethylene diisocyanate (HDI), 3,4-dihydroxy-L-phenylalanine (L-DOPA), zinc chloride (ZnCl_2_), strontium chloride (SrCl_2_) calcium chloride (CaCl_2_), magnesium chloride (MgCl_2_), and cuprous chloride (CuCl₂) were purchased from Aladdin. Stannous octoate was obtained from Macklin. 3-(4,5-dimethylthiazol-2-yl)-2,5-diphenyltetrazolium bromide (MTT) for cytotoxicity test was purchased from Sigma-Aldrich. Fetal bovine serum (FBS) and Dulbecco’s modified eagle medium (DMEM) were obtained from Thermo-Fisher.

## Methods

### Preparation of Adhesives

PEG initiated the ring-opening polymerization of GA and LA in an anhydrous environment using stannous octoate as the catalyst. The reaction was conducted in toluene (25 wt%) under oxygen-free conditions for 8 hours, followed by toluene removal using an oil pump under vacuum. The product was then dissolved in chloroform, precipitated in cold ether, and vacuum-dried to obtain the viscous PLGA-PEG-PLGA copolymer (NG) product.

NG was dissolved in toluene (25 wt%) under a nitrogen atmosphere, and a small amount of HDI (0.5 wt%) and stannous octoate was added. The reaction was maintained at 70 °C for 4 hours. Following the reaction, the product underwent standard post-processing steps, including solvent removal, re-dissolution in chloroform, precipitation in cold ether, and vacuum drying, to yield the chemical crosslinked copolymer adhesive CG.

The L-DOPA-incorporated adhesive CGD was prepared by uniformly dispersing L-DOPA powder into CG at a mass ratio of 2:9 after the precipitation step and before vacuum drying. Varying mass ratios of metal chlorides were mixed with L-DOPA and then uniformly dispersed into CG (**Table S1**).

### Characterization of Adhesives

^1^H NMR spectra were acquired on a Bruker Avance NEO spectrometer (500 MHz, Switzerland) using deuterated chloroform (CDCl₃) as the solvent. Data analysis was performed using MestReNova software. The molecular weights (number-average, Mn; weight-average, Mw) and polydispersity index (PDI = Mw/Mn) of the copolymers were determined by gel permeation chromatography (GPC) with tetrahydrofuran as the mobile phase (flow rate: 1.0 mL/min, temperature: 30 °C). Fourier-transform infrared (FT-IR) spectra were recorded on Nicolet IS50 spectrometer (Thermo Scientific™, USA) using potassium bromide pellets, with wavenumbers ranging from 4000 to 400 cm⁻¹. X-ray diffraction (XRD) patterns were collected on a Rigaku SmartLab diffractometer (Cu-Kα radiation, λ = 0.1542 nm, 40 kV, 30 mA, λ = 0.1542 nm) with a scanning range of 5° to 70° and a sweep speed of 10°/min. Thermogravimetric analysis (TGA) was performed on a TA Q50 instrument (USA) under a nitrogen atmosphere, with a temperature range of 25 to 800 °C and a heating rate of 20 °C/min.

DSC curves were recorded using a TA Instrument (DSC Q2000, USA). The rheological properties of the adhesives were measured using an ARES-G2 strain-controlled rheometer (TA, USA). For the temperature sweep measurement, the sample was loaded at high *T* where the sample can flow (≤100 °C), and reduced to low *T* (≤~ -40 °C) where the high-frequency modulus was ~10^9^ Pa, and the temperature sweep was given from the lowest *T* to the highest *T* at an angular frequency of 1 rad/s, 0.5% initial strain, and a temperature ramp rate of 3 °C/min. The strain amplitude was kept small and within the linear region, and increased slightly with increasing the temperature to maintain the torque to be above the sensitivity of the transducer. The microstructure of freeze-dried adhesives was visualized using cryo-field emission scanning electron microscopy (SEM, Sigma 300, Zeiss, Japan). Water contact angle tests were performed on Hamburg100 droplet shape analyzer (KRÜSS, Germany).

Lap shear strength tests were conducted using an LLOYD universal material testing machine (manufactured by the American company Ametek) on various substrates, including glass, plastic, and aluminum sheets. The overlap area was 25 mm × 25 mm, and the elongation rate was set to 50 mm/min.

The a-o components listed in Table.S1 for MgCl₂ and ZnCl₂, along with the subsequently stronger bonding strengths observed for CaCl₂, SrCl₂, and CuCl₂, were subjected to adhesion size testing. Optimal ratios were determined from these tests and used for subsequent characterizations. Plastic, glass, and aluminum were selected as test substrates, and testing was conducted at time points of 0, 15, 60, and 105 days.

Reversible intermolecular interactions enabled initial repeated adhesion and repeatability testing. In these tests, a pair of substrate sheets (2.5 cm × 2.5 cm) were adhered for 3 seconds, pulled apart, and then re-adhered for another 3 seconds. This cycle was repeated for mechanical testing, with the number of cycles exceeding 120.

### Molecular Dynamics Simulations

All molecular dynamics simulations were carried out using Gromacs 2018.8 program with the General Amber Force Field (GAFF).^1,2^ In this study, all molecules were optimized by Gaussian 16 program using M06-2X hybrid functional with 6-31g* basis set. Multiwfn was used to construct RESP charges.^3^ The Python script of ztop.py, utilized for the generation of forcefield parameters and the construction of polymers, can be found at https://gitee.com/coordmagic/coordmagic/blob/master/ztop.py.

One PLGA-PEG-PLGA molecule, one HDI molecule, 20 L-dopa molecules, 40 Mg²⁺ ions, and 80 Cl⁻ ions (or alternatively, one PLGA-PEG-PLGA molecule and one HDI molecule) were placed into a 4 × 4 × 4 nm simulation box using the Packmol software.^4^ The cut-off for neighbor list of Verlet method and that for short-range interactions is 1.2 nm in all calculations with periodic boundary conditions in all three directions. Following energy minimization, all systems were finally equilibrated in the NVT ensemble for 20 ns at 298.15K. The time step of each simulation was set to be 2 fs. Upon completion of the simulations, the final simulated structures were extracted and placed onto the surfaces of aluminum sheets. The same simulation steps were then repeated for these systems. IGM (Independent Gradient Model) analysis was performed using Multiwfn.^2^ Interaction energy analysis (E_intermolecular-nonbonding-interaction_) was calculated as the sum of Short-range Coulomb interactions (E_Coul-SR_) and Long-range van der Waals interactions (E_LJ-SR_), E_intermolecular-nonbonding-interaction_= E_Coul-SR_ + E_LJ-SR_.

### Sealant Test

A 5 mm diameter leak was punctured at the bottom of mineral water bottles of varying sizes. The bottles were filled with tap water (with blue pigment added for visualization), and the leaks were sealed using CG. Mineral water bottles with different volumes (350 mL, 600 mL, and 3 L) were used to simulate varying pressure conditions. In the fourth panel, six leaks were evenly distributed at 360° around the bottom of the bottle to simulate a scenario with multiple leakage points.

### In Vitro Degradation

Approximately 1 g (Wa) of the sample was placed into a 5 mL centrifuge tube and immersed in 2 mL of PBS buffer solution at 25 °C. At designated time points, the upper layer of the degradation solution was removed for pH measurement. The remaining residue was then freeze-dried and weighed (Wb). The residual amount was calculated using the following equation:

Residual amount (%)=
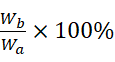


### Biocompatibility of Adhesives

Proliferation of L929 cells co-cultured with the adhesives was evaluated using a standard MTT assay. L929 cells were seeded onto different adhesive groups (control, NG, CG, CGD, CGDMgCl₂, and CGDZnCl₂) at a density of 1.0 × 10⁵ cells/mL and cultured in vitro for 24 hours. A live/dead cell viability assay was also performed on the L929 cells, and images were captured using a Nikon ECLIPSE/Ti SERIES microscope (Tokyo, Japan).

### Statistical Analysis

All experiments were performed in triplicate, and statistical analysis was conducted using Origin 2020 software. For all tests, statistical significance was denoted as follows: *p < 0.05, **p < 0.01, and ***p < 0.001.

### References

(1) Abraham, M. J.; Murtola, T.; Schulz, R.; Páll, S.; Smith, J. C.; Hess, B.; Lindahl, E. GROMACS: High Performance Molecular Simulations through Multi-Level Parallelism from Laptops to Supercomputers. *SoftwareX* **2015**, *1-2*, 19-25.

(2) Wang, J.; Wolf, R. M.; Caldwell, J. W.; Kollman, P. A.; Case, D. A. Development and Testing of A General Amber Force Field. *J. Comput. Chem.* **2004**, *25* (9), 1157-1174.

(3) Lu, T.; Chen, F. Multiwfn: A Multifunctional Wavefunction Analyzer. *J. Comput. Chem.* **2012**, *33* (5), 580-592.

(4) Martínez, L.; Andrade, R.; Birgin, E. G.; Martínez, J. M. PACKMOL: A Package for Building Initial Configurations for Molecular Dynamics Simulations. *J. Comput. Chem.* **2009**, *30* (13), 2157-2164.

# Supplementary Figures and Tables

**70℃**

chelation

crosslinking

hydrogen bond

**Figure S1.** Schematic illustration. Preparation of copolymer matrix and the interactions involved in composite adhesive.


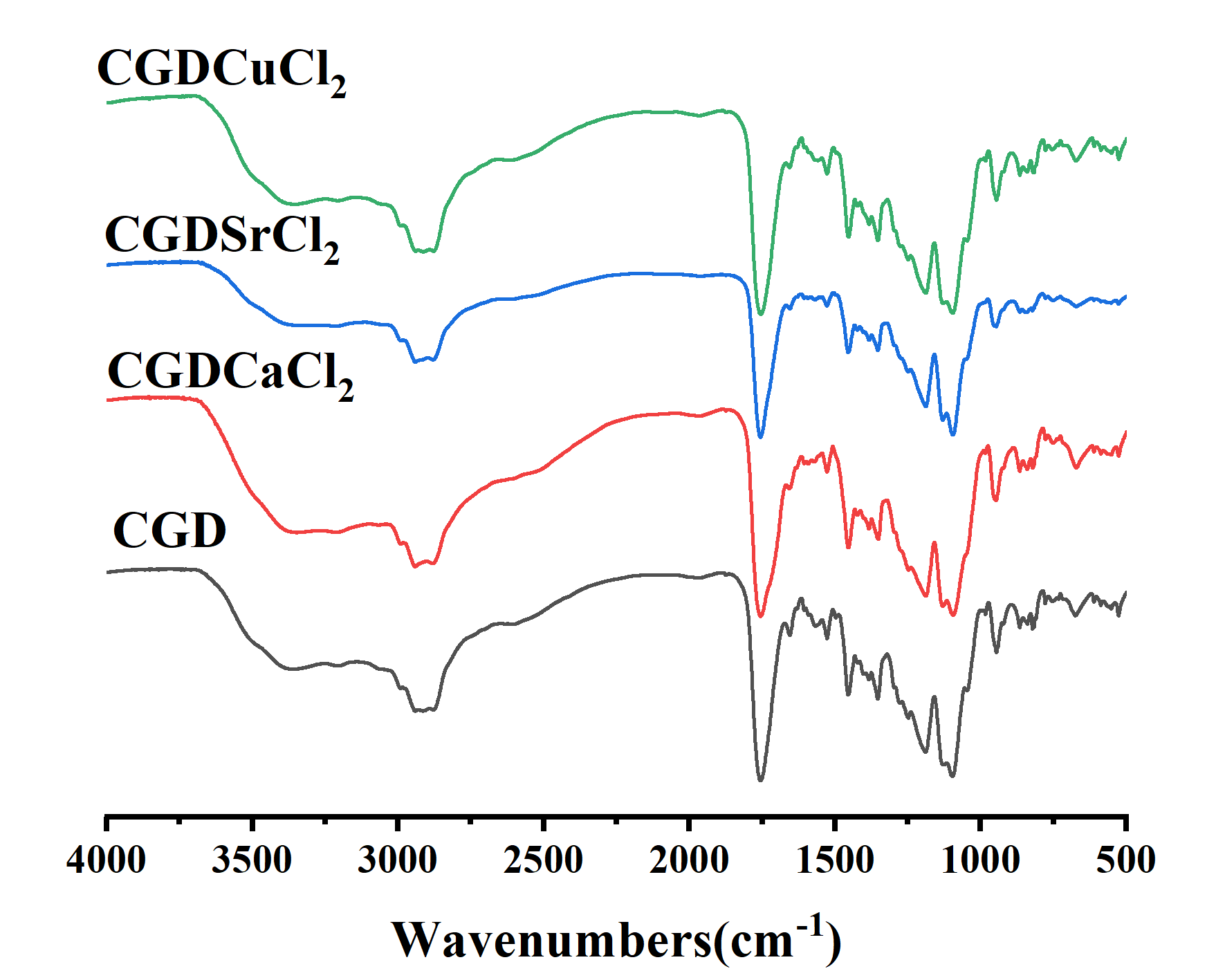
**Figure S2.** FT-IR spectra of adhesives.


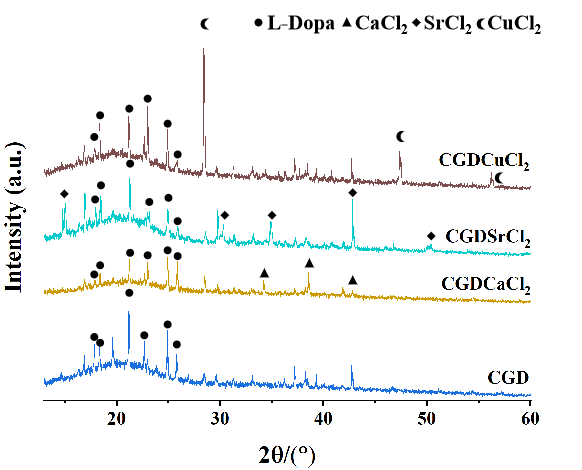
**Figure S3.** XRD patterns of adhesives.


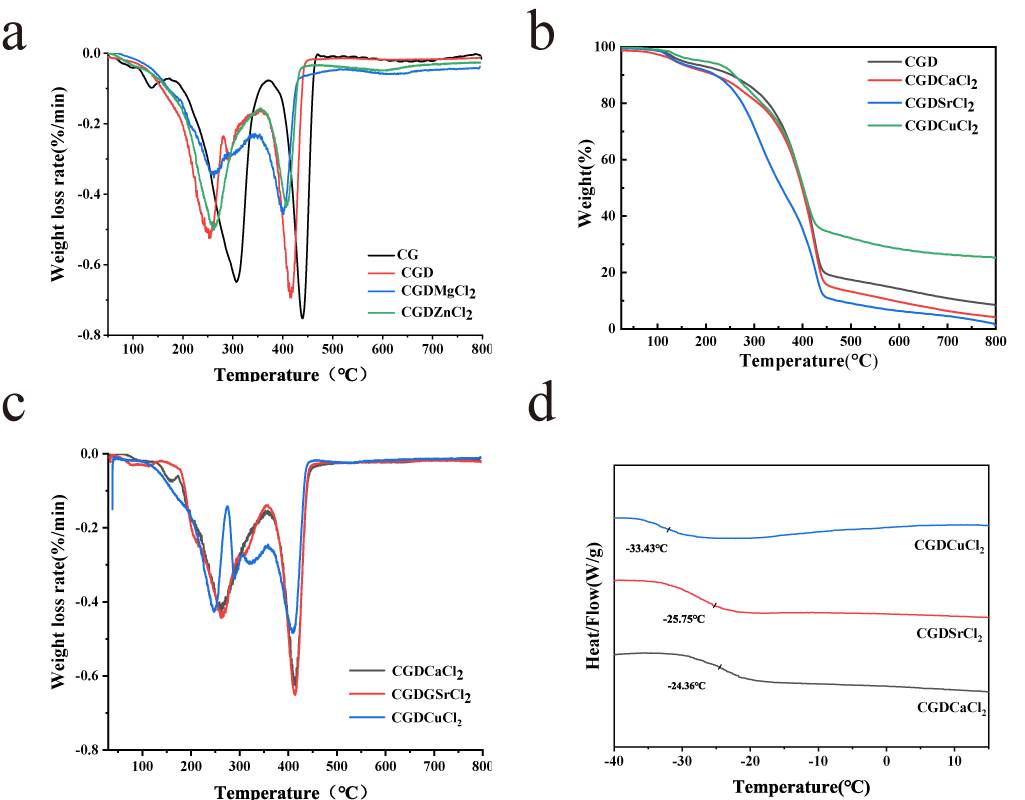
**Figure S4.** Thermal properties characterization. (a) DTG profiles of CG, CGD, CGDMgCl_2_ and CGDZnCl_2_. (b-d) TGA, DTG and DSC curves of CGDCuCl_2_, CGDSrCl_2_, and CGDMgCl_2_.


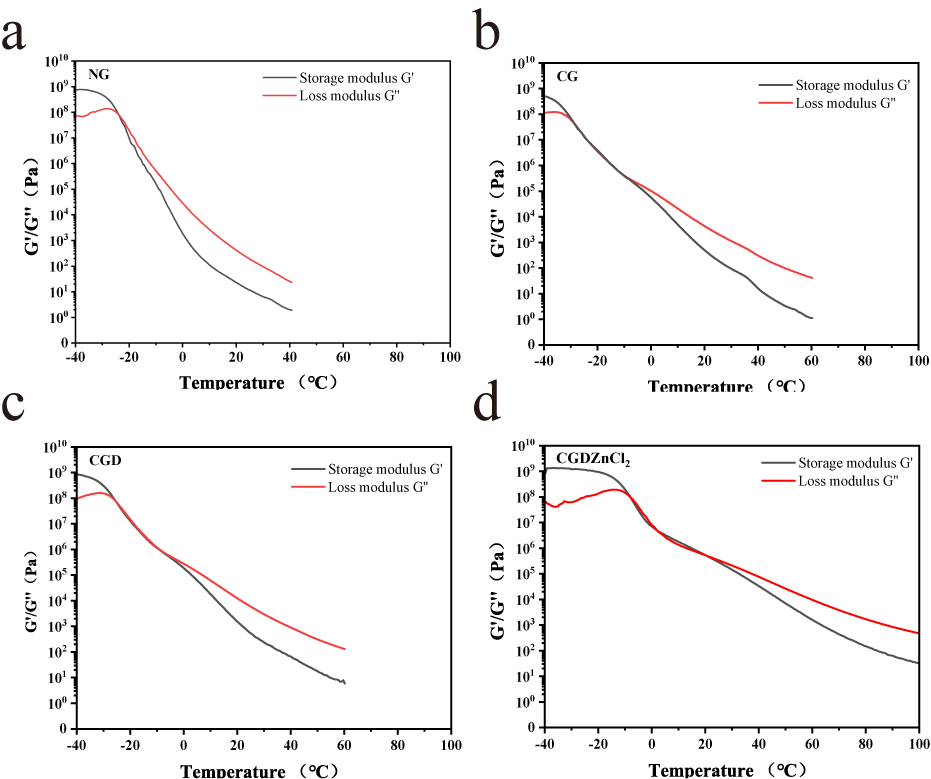
**Figure S5.** Storage modulus and loss modulus of the NG、CG、CGD and CGDZnCl_2_ at different temperatures.

**CGDCaCl_2_**

**CGDSrCl_2_**

**CGDCuCl_2_**

**Figure S6.** Water contact angle measurements of CGDCaCl_2_, CGDSrCl_2_, and CGDCuCl_2_ adhesives.


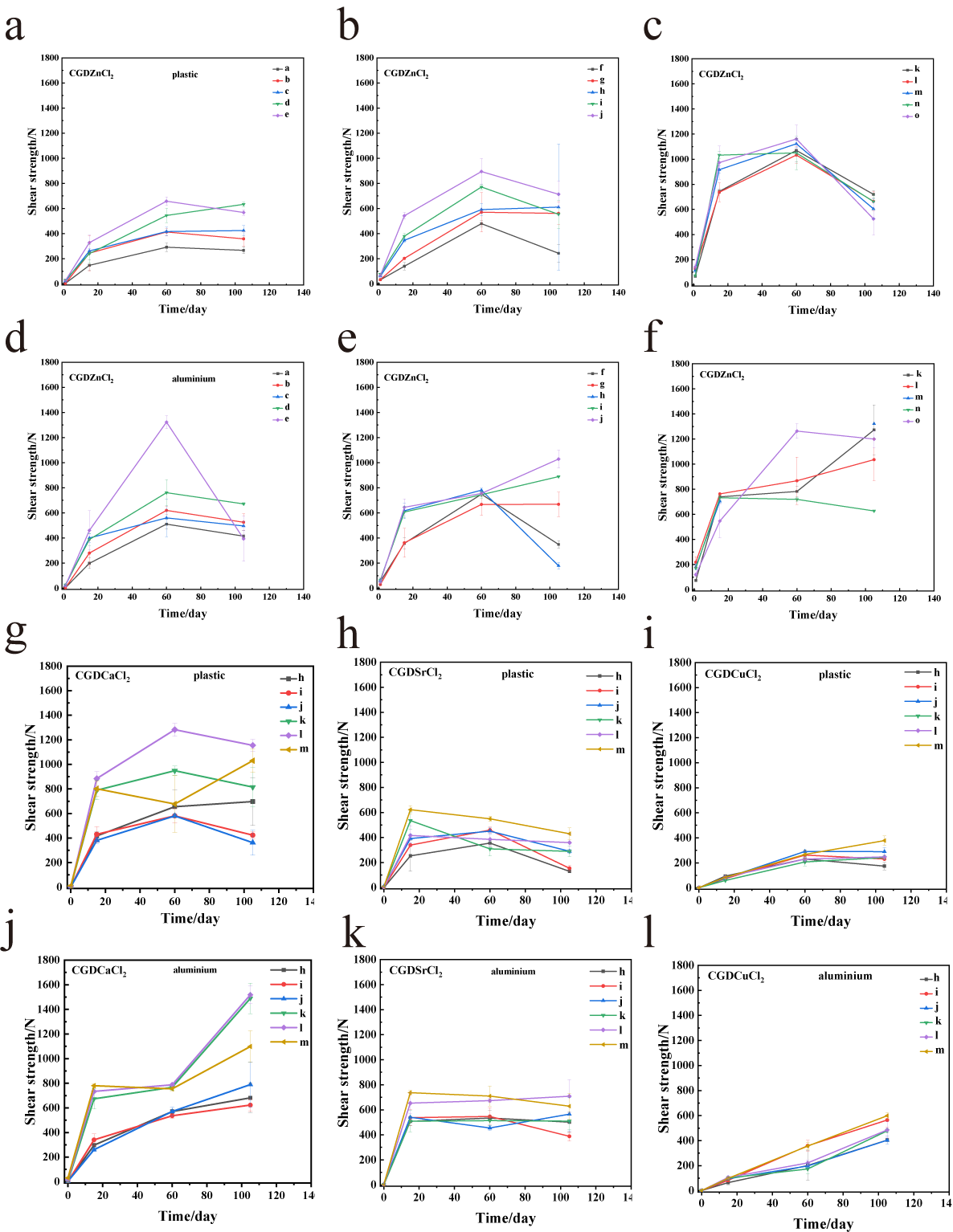


**Figure S7.** (a-f) Shear strength of the a-o component of CGDZnCl_2_ on different materials (a-c on plastic, d-f, on aluminum). (g-l) Shear strength of the a-o component of CGDZnCl_2_, SrCl_2_, CuCl_2_ on different materials (plastic, aluminum).


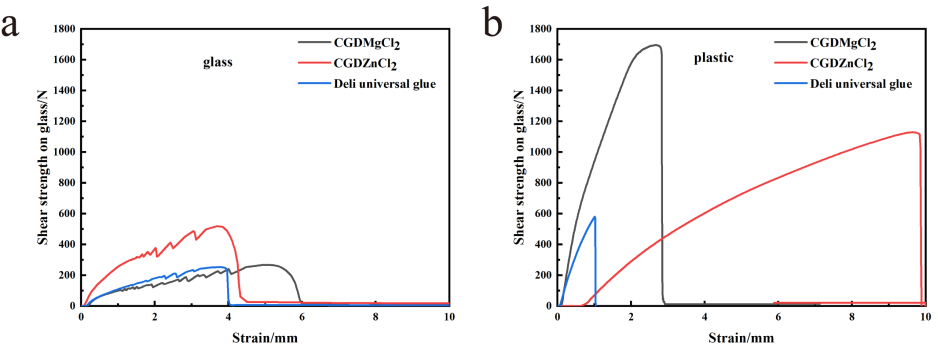
**Figure S8.** Comparison of shear strength: (a) on glass. (b) on plastic among CGDMgCl_2_, CGDZnCl_2_ and Deli Universal glue.


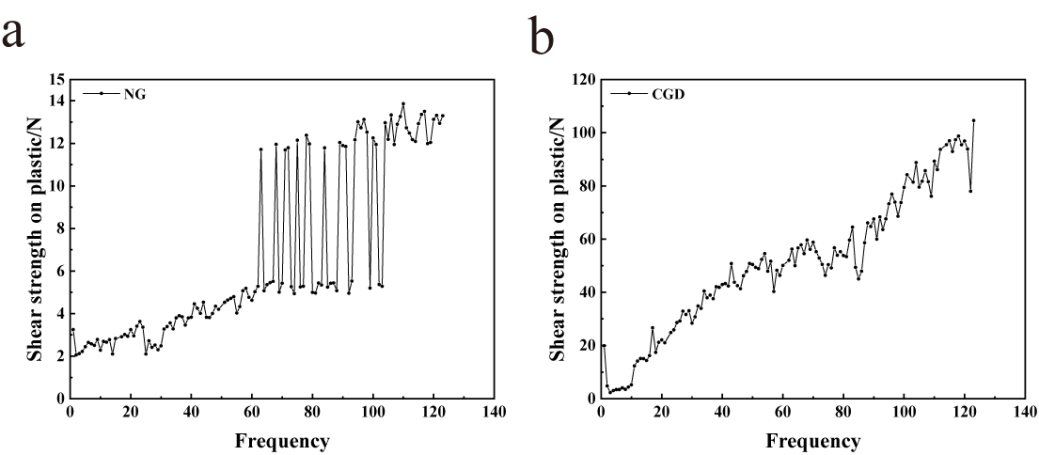


**Figure S9.** Change trend in shear strength upon repeated bonding (up to 120 cycles) to plastic substrates: (a) NG. (b) CGD.


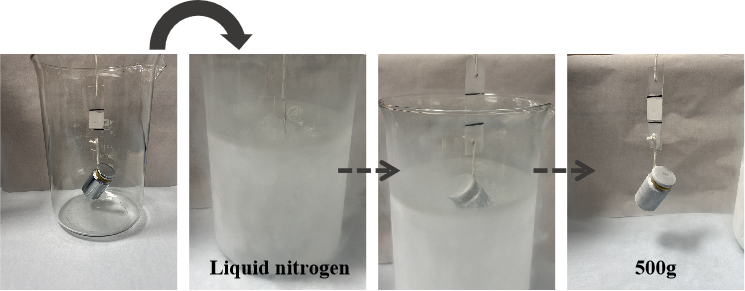
**Figure S10.** CGDMgCl_2_ maintained strong adhesion, sustaining a 500 g load throughout the entire nitrogen immersion cycle.

**Figure S11.** Sealant effect evaluation. (a) Instant sealing of leaks in plastic cups (right). (b) Immediate sealing capability for 50 mL plastic centrifuge tubes (right). (c) Long-term sealing performance at various temperatures (4°C, 12°C, 25°C) and pressures (simulated by different water bottle volumes) after 1 year. (d) The water bottle with uniformly distributed leakage points at the base maintained complete sealing after 1 year.


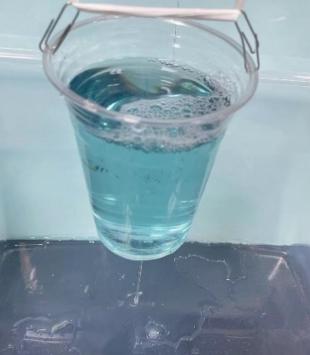

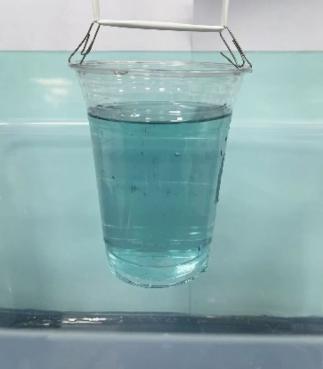

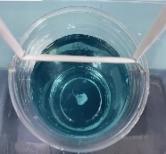

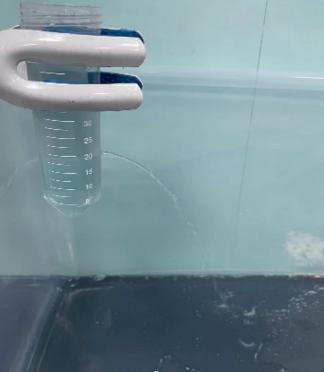

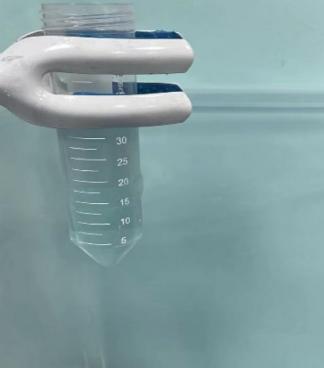


**a**

**b**

**c**

**d**


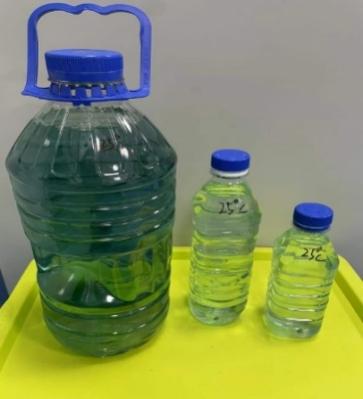

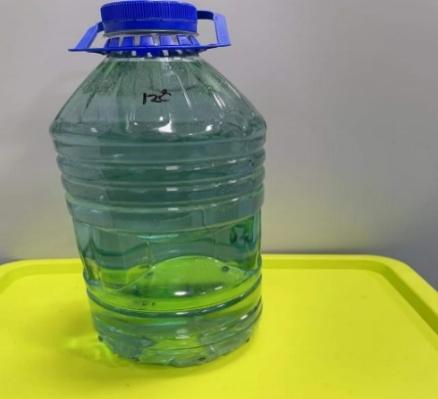

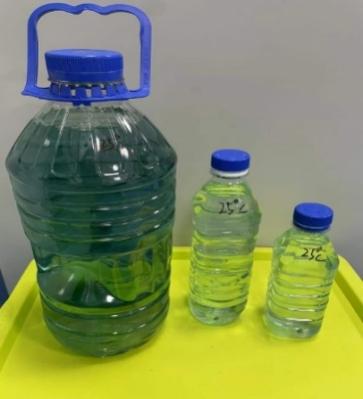

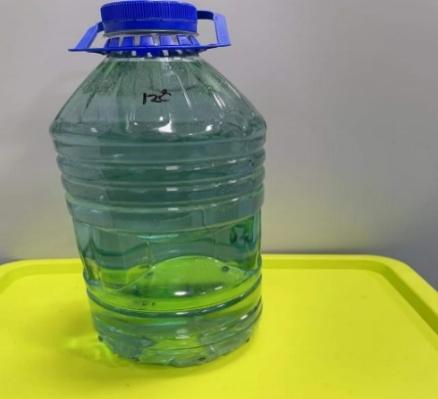

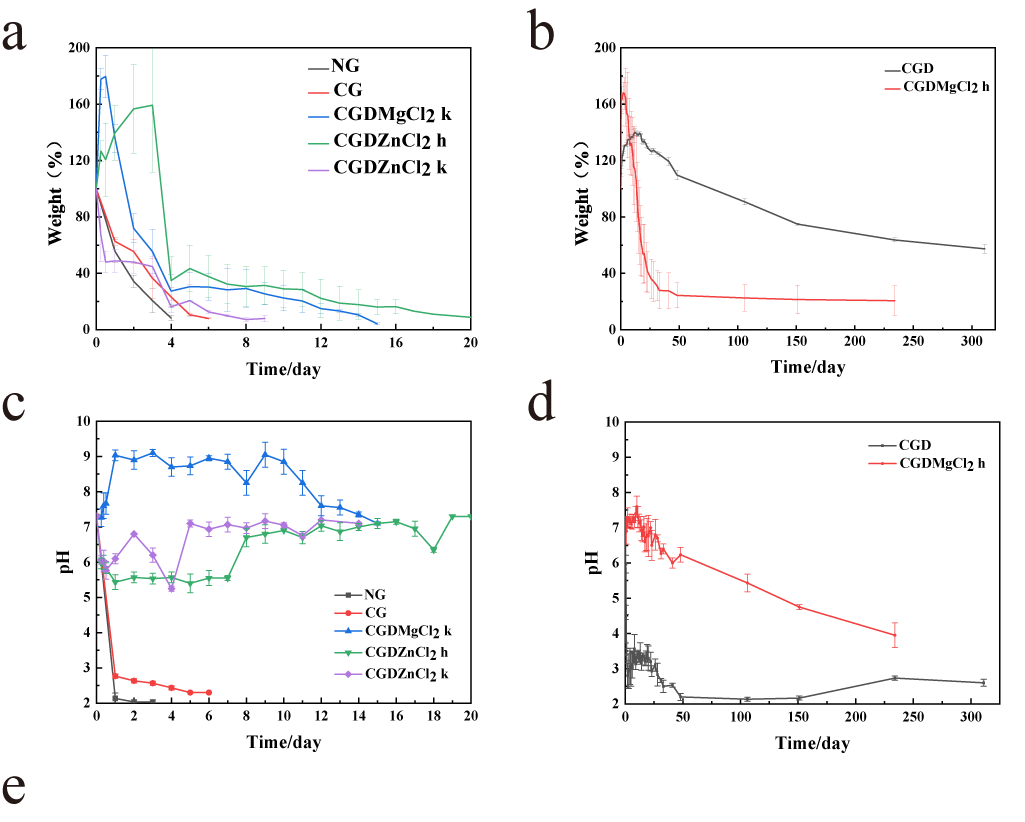


**drop off**

**5 s**


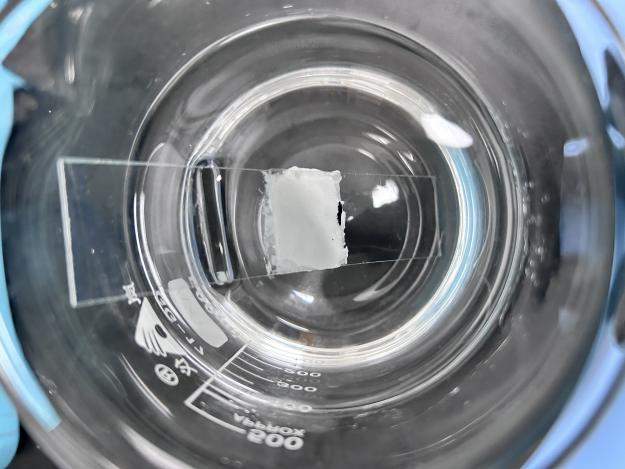

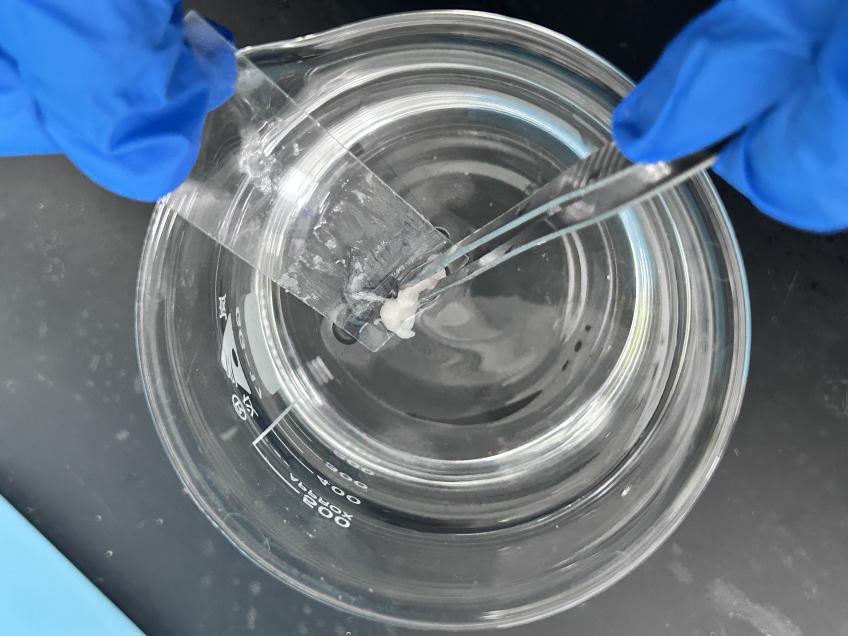


**Figure S12.** In vitro degradation of adhesives. (a, b) Residual weight ratio of the adhesives during degradation. (c, d) pH measurement for each adhesive group. (e) The bonded glass substrates detached after 5 h of alcohol immersion.


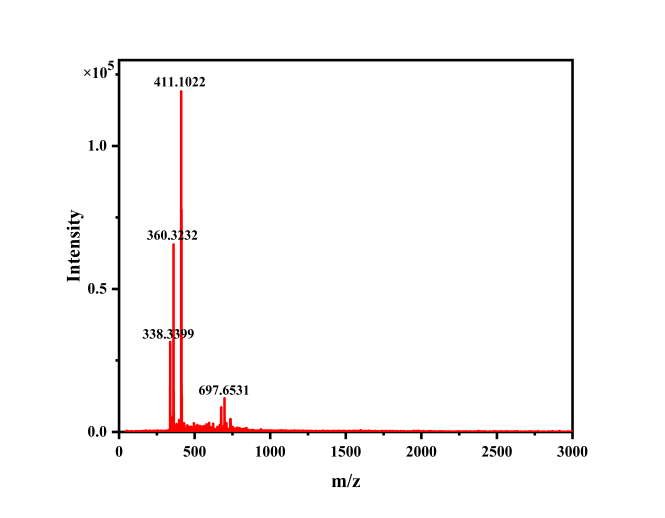
**Figure S13.** Mass spectrum of degraded CG.

**Figure S14.** Cell availability assessment. (a-c) Cell viability measured by MTT assay at 24 h, 48 h, and 72 h. (d) Live/dead staining at 24 h: viable cells (green) and dead cells (red).


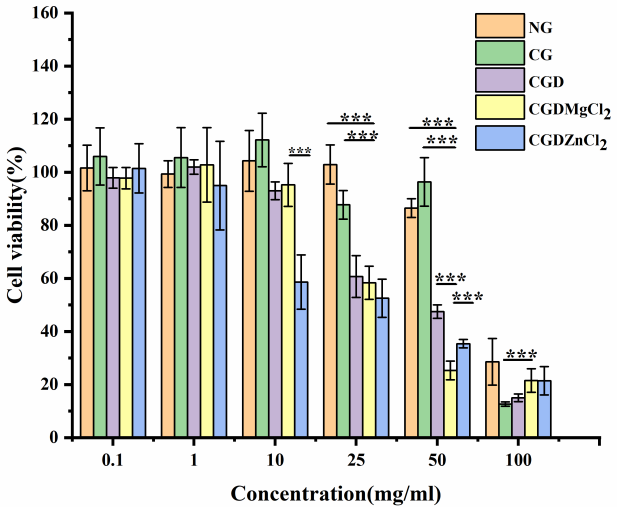


**a**

**b**


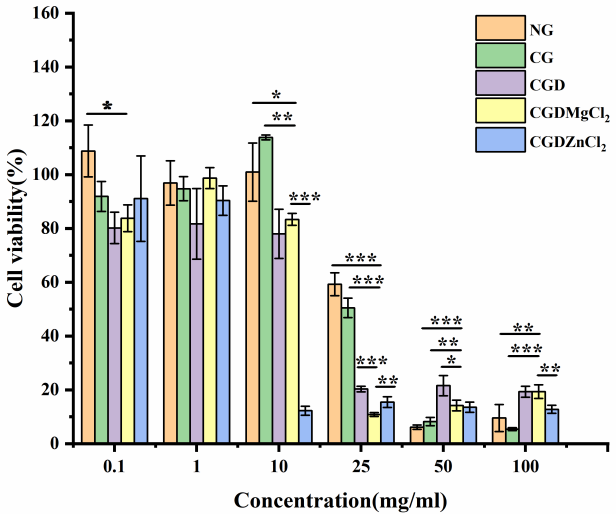

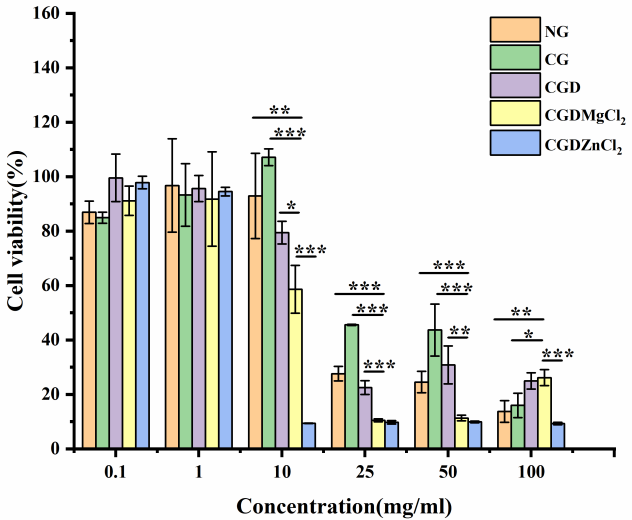


**c**


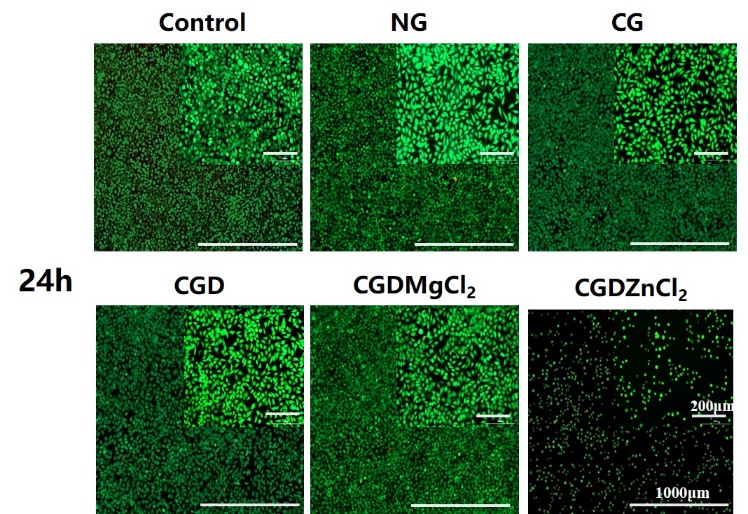


**d**

**Table S1.** Group Proportioning

| Components | l-DOPA/g | Metal Chloride/g |
| --- | --- | --- |
| a-e | 1 | 0.5、1、2、3、4 |
| f-j | 2 | 0.5、1、2、3、4 |
| k-o | 4 | 0.5、1、2、3、4 |

|  | -30℃ | | 0℃ | | 25℃ | | | T_g,dsc_  （℃） | Temp. in max（℃） | |
| --- | --- | --- | --- | --- | --- | --- | --- | --- | --- | --- |
|  | *G'*（MPa） | Viscosity(Pa·s) | *G*' | Viscosity | *G'* | Viscosity | υ_e_ （mol•m^-3^） |  | Peak1 | Peak2 |
| NG | 489.7 | 5.06×10^8^ | 0.002 | 2.47×10^4^ | 1.68×10^-5^ | 1.89×10^2^ | 0.0068 | -22.95 | -17.95 | 8.747 |
| CG | 82.6 | 1.08×10^8^ | 0.05 | 1.05×10^5^ | 1.96×10^-4^ | 2.13×10^3^ | 0.079 | -25.25 | -27.36 | 28.21 |
| CGD | 313.3 | 3.48×10^8^ | 0.17 | 3.05×10^5^ | 5.31×10^-4^ | 5.88×10^3^ | 0.21 | -16.27 | -23.25 | 31.38 |
| CGD  MgCl_2_ | 1023.3 | 1.02×10^9^ | 6.06 | 9.34×10^6^ | 0.43 | 5.60×10^5^ | 173.56 | -11.54 | -2.87 | >100 |
| CGD  ZnCl_2_ | 1205.6 | 1.28×10^9^ | 6.39 | 9.61×10^6^ | 0.27 | 4.20×10^5^ | 108.98 | -11.11 | -3.20 | >100 |

**Table S2.** Rheological Data.

# Movie Descriptions

**Movie S1.** The adhered plastic sheet (overlap area: 2.5 cm × 2.5 cm) successfully lifted a 10 kg bucket.

**Movie S2.** The plastic adhesive joint stably maintained a 500 g load while fully immersed in liquid nitrogen.
